# Dynamic High-Pressure Microfluidization-Treated Pectin under Different Ethanol Concentrations

**DOI:** 10.3390/polym10121410

**Published:** 2018-12-19

**Authors:** Cheng-Mei Liu, Lu Liang, Xi-Xiang Shuai, Rui-Hong Liang, Jun Chen

**Affiliations:** State Key Laboratory of Food Science and Technology, Nanchang University, 235 Nanjing East Road, Nanchang 330047, China; liuchengmei@ncu.edu.cn (C.-M.L.); 18679113010@163.com (L.L.); shuaixixiang1989@163.com (X.-X.S.); liangruihong@ncu.edu.cn (R.-H.L.)

**Keywords:** dynamic high-pressure microfluidization, pectin, ethanol, disaggregation, degradation

## Abstract

We previously reported that dynamic high-pressure microfluidization (DHPM) can degrade pectin in aqueous solution. In this study, we further investigated the effect of DHPM on pectin in water-ethanol systems. In the absence of DHPM treatment, it was found that pectin exhibited increased average particle size and unchanged average molecular weight, but a decline in reducing-sugar-ends content with the increase of ethanol concentrations (0–10% *v*/*v*). These results indicated that the addition of ethanol induced aggregation of pectin. During DHPM treatment, pectin underwent disaggregation and degradation under all measured ethanol concentrations. Disaggregation was enhanced but degradation was weakened with the increase of ethanol concentration. FT-IR and UV spectra indicated that demethylation but no β-elimination occurred in the water-ethanol system during DHPM. Finally, the mechanism of DHPM-induced disaggregation and degradation of pectin under a water-ethanol system was updated. This work may help us to find a suitable condition for reducing the degradation of pectin during the process of homogenization.

## 1. Introduction

Pectin, a main structural component of plant cell walls, is a complex polysaccharide that is composed of a backbone of (1→4)-linked α-d-galacturonic acid units. It is widely used in the food industry as a gelling agent, thickener, texturizer, emulsifier and stabilizer to modify food properties [1,2,3]. One of the greatest difficulties in its optimal utilization is the tendency to undergo degradation caused by various treatments, such as acid hydrolysis [4], thermal processing [5,6], enzymatic degradation [7] and some mechanical treatments [8,9].

Dynamic high-pressure microfluidization (DHPM) is a new-type high-pressure homogenization technology. It combines forces of high-velocity impact, high-frequency vibration, instantaneous pressure drop, cavitation, intense shear and ultra-high pressures up to 200 MPa with a short treatment time (less than 5 s) [10,11]. This technology is highly effective for modification of food macromolecules. The structures and properties of enzymes [11], nonenzymatic proteins [12], starch [13], nonstarch polysaccharides [14,15] and dietary fiber [16] have been modified by using DHPM.

Zhang et al. [10] studied the effects of DHPM on characteristics of pectin extracted from black-cherry tomato waste, and found that DHPM caused an effective increase of the average particle size, but a significant decrease of the apparent viscosity of pectin. Xie et al. [17] studied the effects of DHPM on potato peel waste pectin, and found that DHPM caused an increase of the galacturonic acid contents, viscosity and emulsifying properties, but a decrease of *M*_w_. Our previous study also reported the degradation of high-methoxyl pectin induced by DHPM, and proposed that part of the DHPM-supplied energy sheared or tore open long pectin chains by a combination of powerful shear, high-velocity impact and high-frequency vibration. The opening of polymer chains exposed a high number of bonds to mechanical forces and H^+^ attack. This effect thus increased the susceptibility of pectin to DHPM-induced degradation [18]. Shpigelman et al. [19] also suggested that the tendency of pectin to undergo depolymerization or degradation due to high-pressure homogenization was dependent on the different pectin conformations in solution, which means the effect of pectin conformation in solution cannot be ignored. Hence, pectin with various aggregation states (conformations) may undergo different extents of degradation during DHPM. However, this hypothesis requires further verification. Preparation of pectin with different aggregation states is the key step to prove the hypothesis.

Generally, the aggregation states (conformations) of polysaccharides can be modified by changing ionic strength [20,21] or adding organic solvents [22]. However, the ions added into the solution are difficult to remove from the solvent, and their presence could affect the subsequent analysis. By contrast, added organic solvents such as methanol, ethanol, chloroform and acetone can be easily removed through evaporation.

In this study, pectin was first dispersed in water-ethanol solutions with different ethanol concentrations and then subjected to DHPM. Subsequently, the average particle size, average molecular weight (*M*_w_), reducing-sugar-ends content, surface topography and possible reactions of pectin were evaluated to elucidate the mechanism underlying DHPM-induced pectin degradation or disaggregation in the water-ethanol system. This work may help us to find a suitable condition for reducing the degradation of pectin during the process of homogenization.

## 2. Material and Methods

### 2.1. Materials and Chemicals

Citrus pectin was purchased from Sigma Aldrich (P9135, Shanghai, China). Prior to use, pectin was purified in accordance with the method of Yapo et al. [23]. Samples were dissolved in deionized water, and centrifuged (RC-5C Plus centrifuge, Sorvall, Norwalk, CT, USA) at 30,000 g for 20 min to remove the water-insoluble fraction. The supernatants were filtered through 3-mm Millipore membranes (Millipore Co., Milford, MA, USA) and freeze-dried (Labconco, Kansas City, MO, USA). The galacturonic acid content of purified pectin was 81.2% as determined by the method of Blumenkrantz et al. [24]. The degree of methoxylation was 67.1% as determined by the method of Food Chemical Codex [25]. All reagents used in this experiment were of analytical grade.

### 2.2. Preparation of Pectin Solution and DHPM Treatment

Purified citrus pectin was dispersed in water-ethanol solution at room temperature to a concentration of 2.0 mg/mL. The ethanol concentrations used were 0%, 2.0%, 4.0%, 8.0% and 10.0% (*v*/*v*). DHPM was performed using an M-100EH-30 microfluidizer (Microfluidics Co., Newton, MA, USA). The dispersions were subjected to five passes of DHPM at 160 MPa. During DHPM treatment, the initial inlet volume of the suspension was 700 mL, and 200 mL suspension was received finally after five passes, because the initial 50 mL and the final 50 mL suspensions of each pass were discarded to prevent the dilution effect during the process. The temperature was controlled at 20–25 °C by covering the reaction chamber and outlet pipe with ice. It took about 180 s for each sample to finish each pass. Pectin solutions prepared with different ethanol concentrations but without DHPM treatment were used as controls. Aliquots of the solutions were collected for determination of average particle size, average *M*_w_, reducing-sugar-ends content analysis and ultraviolet (UV) spectroscopy. The remaining dispersions were lyophilized for surface morphology analysis and FT-IR.

### 2.3. Characterization

#### 2.3.1. Determination of Average Particle Size

Pectin dispersions were diluted with the corresponding solvent to a concentration of 0.5 mg/mL, then the average particle sizes were determined through the dynamic light scattering with a Nicomp 380/ZLS Zeta potential/particle sizer (PSS Nicomp, Santa Barbara, CA, USA). All measurements were performed at 25 °C [26].

#### 2.3.2. Determination of Average *M*_w_

The average *M*_w_ was determined with an Agilent high-performance size-exclusion chromatography (HPSEC) system, which comprised an Agilent 1200 pump unit, an automatic injector (Agilent Technologies, Waldbroon, Germany) and a refractive index detector (Brookhaven Inc., New York, NY, USA). Samples were diluted with the corresponding solvent to a concentration of 0.5 mg/mL and then filtered through 0.45 mm filters. The analysis was conducted with a PL aquagel-OH MIX column (300 mm × 7.5 mm, 8 μm) and eluted at 0.7 mL/min. The mobile phase consisted of 0.05 M NaNO_3_ with 0.2 g/L Na_3_N as a preservative [27]. Dextran standards (T-2000, T-500, T-70, T-40 and T-10, Sigma Aldrich, Shanghai, China) and glucose (*M*_w_ 180, Sigma Aldrich, Shanghai, China) were used to calibrate the column and construct a standard curve.

#### 2.3.3. Determination of Reducing-Sugar-Ends Content

The reducing-sugar-ends content was determined by the dinitrosalicylic acid (DNS) method [28] with minor modifications. In brief, 2 mL of sample was mixed with 1.5 mL of modified DNS reagent, which consisted of 1% DNS, 0.2% phenol, 0.5% sodium sulfite and 1% sodium hydroxide. The mixture then was heated for 5 min at 100 °C. Prior to cooling, roselle salt solution (1 mL, 40%) was added to the mixture for color development. Finally, the samples were subjected to UV/Vis spectrophotometry (UV-2500, Shimadzu, Kyoto, Japan) at the wavelength of 540 nm. Calibration curves were established on the basis of different galacturonic acid concentrations.

#### 2.3.4. Surface Morphology

DHPM-treated pectins and the controls were lyophilized, then visualized with an environmental scanning electron microscope (Quanta200F, FEI Deutschland GmbH, Kassel, Germany) in low-vacuum mode and operated at 30 kV voltage and 3.0 spot size. Freeze-dried pectin was attached to a circular specimen stub by using double-sided adhesive tape [18].

#### 2.3.5. Investigation of the Possible Reactions on Pectin Induced by DHPM

To quantify the extent of β-elimination of samples, pectin solutions were transferred to ultraviolet (UV) quartz cuvettes and analyzed using a UV spectrophotometer (UV-2500, Shimadzu, Japan) at 25 °C. Spectra were obtained over the wavelength range of 200–400 nm [29].

The FT-IR spectra of pectin were obtained using a Nicolet 5700 spectrometer (Thermo Co., Madison, WI, USA). Dried samples were ground with spectroscopic-grade KBr powder (Sigma Aldrich, Shanghai, China) and pressed into pellets for spectral measurement over the frequency range of 4000–400 cm^−1^ with the resolution of 2 cm^−1^ and 32 scans. The data were collected and plotted as transmittance (%) as a function of the wavenumber (cm^−1^) and analyzed using Ominic 7.2 software (Spectra-Tech Inc., Madison, WI, USA) [30].

The degree of methoxylation (DM) was calculated using FT-IR spectra based on the method of Gnanasambandam et al. [31]; a linear correlation was established between the ratio of the peak area at 1740 cm^−1^ (υ_(C__=O)COOMe_) over the sum of the peak areas at 1740 and 1630 cm^−1^ (υ_as (COO)_) based on pectin standards of known DM, after which the peak area of the ester carbonyl of the sample was fit to the curve to calculate its DM.

### 2.4. Statistical Analysis

All experiments were conducted in triplicate. Statistical analysis was conducted with SPSS (version 16.0, Chicago, IL, USA). Results were expressed as mean ± standard deviations and compared through Tukey’s test with 5% confidence level.

## 3. Results and Discussion

### 3.1. Characterization of Disaggregation and Degradation

#### 3.1.1. Change of Average Particle Size

**Figure 1:**
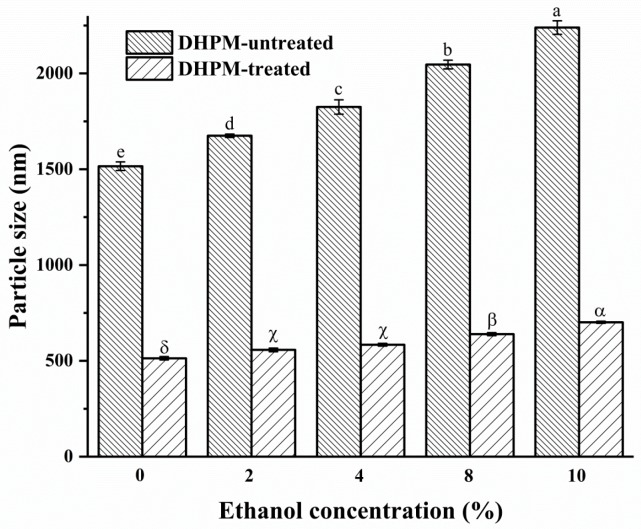
Average particle size of pectin treated with or without DHPM under different ethanol concentrations (error bars represent the standard deviation of three replicates). Different letters (a, b, c,…) for DHPM-untreated samples indicate significant differences (Tukey’s test, *p* < 0.05). Different Greek letters (α, β, γ,…) for DHPM-treated samples indicate significant differences (Tukey’s test, *p* < 0.05).

The effect of ethanol concentration and DHPM on the average particle size of pectin is illustrated in Figure 1. Dynamic laser light scattering results showed that pectin aggregates existed in the samples. Apple pectin has been sized at 0.45 μm [32], and citrus cloud at 400–5000 nm [33]. In our study, DHPM-untreated pectin in aqueous solution consisted of large particles with an average particle size of 1516 (±22) nm. These large particles were considered as polysaccharide aggregates. The tendency of aggregate formation for pectin has been extensively reported [34]. As far as this study was concerned, when the ethanol concentration increased from 0% to 10.0% *v/v*, the average particle size of pectin was found to increase from 1516 (±22) nm to 2240 (±36) nm. The increase of average particle size may be because the increasing ethanol concentration caused a greater extent of aggregation. It was reported that ethanol can compete with pectin in binding water molecules, thus pectin molecules came closer to each other and formed more hydrogen bonds among themselves, which led to a greater extent of aggregation, and even caused the precipitation of pectin with the increase of ethanol concentration [21]. Rimada et al. [35] reported that the addition of large quantities of ethanol in kefiran solutions caused the decrease of kefiran solubility due to binding of large numbers of water molecules by the ethanol, resulting in the formation of kefiran aggregates which increased average particle size.

After DHPM treatment, the average particle size of pectin was significantly reduced as compared to the untreated pectin. The higher the concentration of ethanol, the more significant the decrease of average particle size. The sizes of DHPM-treated pectin were 513.5 (±9.1) nm, 557.3 (±9.3) nm, 584.0 (±7.3) nm, 639.6 (±7.9) nm and 701.3 (±5.5) nm for pectin dispersed at 0%, 2%, 4%, 8% and 10% of ethanol concentration, respectively. Values of particle size conformed to the principle that particles are prone to gather together while particle size is lower than 1–100 μm [36]. However, it is unknown whether the decrease in the average particle size was attributed to either disaggregation or degradation. Therefore, the following characterizations were carried out.

#### 3.1.2. Change of Average *M*_w_

**Figure 2:**
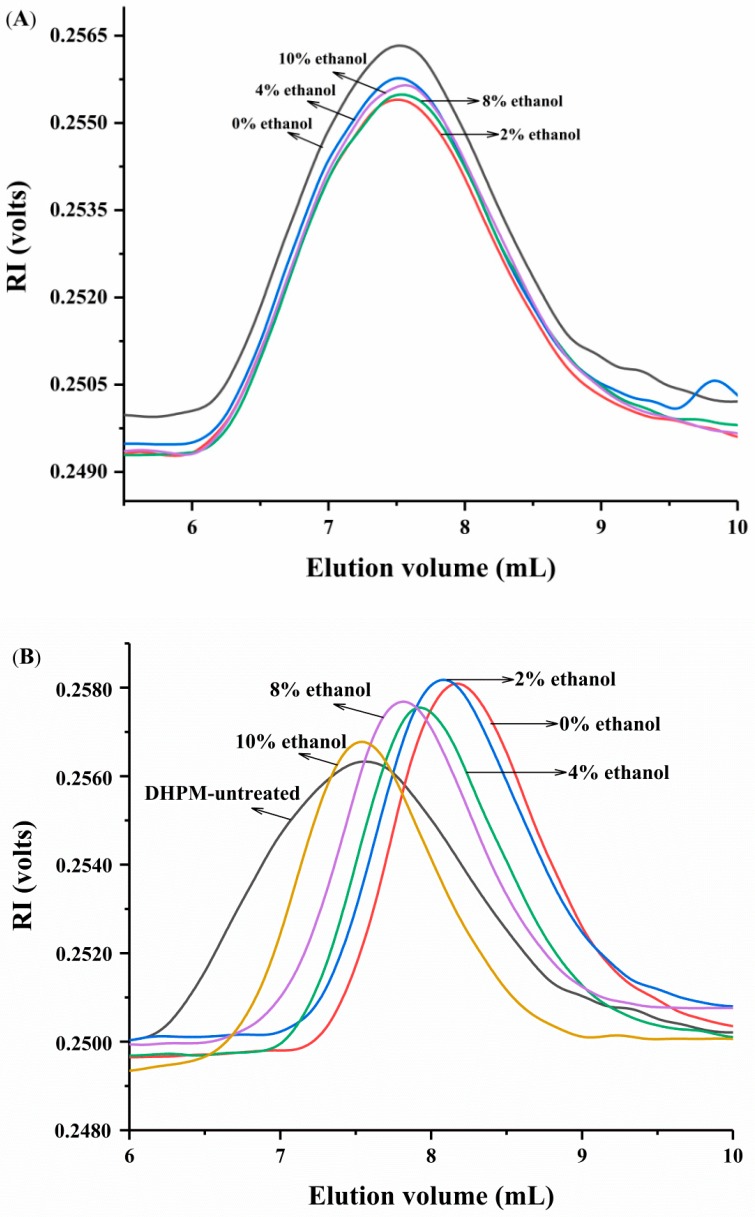
Average *M*_w_ of pectin treated with (**B**) or without (**A**) DHPM under different ethanol concentrations.

The effect of ethanol concentration and DHPM on the average *M*_w_ of pectin is illustrated in Figure 2. The elution volume of DHPM-untreated pectin remained unchanged with the increase of ethanol concentration (Figure 2A). This finding suggested that the average *M*_w_ of pectin did not change despite the addition of ethanol. In addition, it was indicated that ethanol has no effect on *M*_w_ or conformational change of pectin during HPSEC determination, which may be because ethanol was diluted by the mobile phase, resulting in ethanol exerting an insignificant effect on the conformation of pectin in the HPSEC determination. Exarhopoulos et al. [21] studied the conformational characteristics of kefiran in a series of solutes and found that kefiran aggregated in ethanol solution. On the basis of the average *M*_w_ remaining unchanged and the average particle size increasing with increasing ethanol concentrations (Figure 1), it was illustrated that ethanol induced pectin aggregation, which has contributed to the industrial use of ethanol for the precipitation of pectin.

The elution volume of DHPM-treated pectin was increased compared to DHPM-untreated pectin (Figure 2B). The magnitude of increase followed the order of 10% < 8% < 4% < 2% < 0% ethanol concentration. The average *M*_w_ of DHPM-untreated pectin was about 177.8 kDa, and the average *M*_w_ of DHPM-treated pectin was 99.0 kDa, 115.6 kDa, 133.1 kDa, 174.0 kDa and 177.3 kDa in the presence of 0%, 2.0%, 4.0%, 6.0%, 8.0% and 10.0% ethanol, respectively. Compared with the results of the DHPM-treated pectin in aqueous solution, Xie et al. [17] reported that DHPM reduced the *M*_w_ of potato peel pectin from 18.2 × 10^5^ Da to 6.0 × 10^5^ Da at 200 MPa. Chen et al. [18] stated that the *M*_w_ of apple pectin was reduced from 356.0 kDa to 108.3 kDa by DHPM treatment at 160 MPa. In our study, the extent of reduction decreased with the increase of ethanol concentration, which was opposite to the trend of average particle size. Distinguishing whether the changes of average particle size and average *M*_w_ resulted from molecule degradation or aggregate disruption is difficult. The decrease of average *M*_w_ was explained by the breakdown of covalent bonds inside the polymer chain when Floury et al. [27] used an ultra-pressure homogenization to degrade methylcellulose. Meanwhile, Al-Assaf et al. [37] reported that the reduction in the average *M*_w_ of gum may be caused by hydrophobic bond disruption (disaggregation). Measuring the change of reducing-sugar-ends content was reported as a solution to investigate the true reason of average *M*_w_ decrease [38], because glycoside bond breakage can lead to the accumulation of reducing sugar ends [6].

#### 3.1.3. Change of Reducing-Sugar-Ends Content

**Figure 3:**
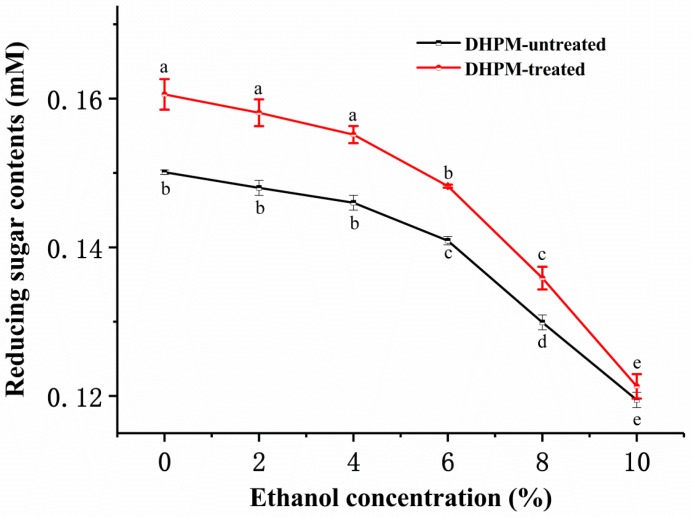
Effects of ethanol concentration and DHPM on reducing-sugar-ends content of pectin (error bars represent the standard deviation of three replicates). Different letters denote significant differences (Tukey’s test, *p* < 0.05).

It is well known that monosaccharides of the pectin link with each other through glycosidic bonds. The reducing sugar ends increase with the breakdown of glycosidic bonds. Theoretically, ethanol addition is not supposed to break down glycosidic bonds and increase reducing-sugar-ends content, because the disruption of glycosidic bonds is mainly caused by β-elimination, acid hydrolysis and mechanical forces [18]. However, in this study, we found that reducing-sugar-ends content decreased with the increase of ethanol concentration (Figure 3). This abnormal phenomenon may be due to precipitation caused by pectin aggregation, which reduced the solute in the solution. The aggregation and precipitation of pectin was enhanced with the increase of ethanol concentration. Hence, the number of detected reducing sugar ends decreased.

It can be found that the reducing-sugar-ends content of pectin increased after DHPM treatment. Without the addition of ethanol, reducing-sugar-ends content was significantly decreased (*p* < 0.05) after DHPM treatment. When the ethanol concentration increased, the relative decrease of reducing-sugar-ends content was decreased with increasing ethanol concentration, indicating that the degradation of pectin induced by DHPM weakened with the rise in ethanol concentration. For example, the amount of reducing sugar ends increased by 7.0%, 6.8%, 6.3%, 5.2%, 4.6% and 1.5% in the presence of 0%, 2.0%, 4.0%, 6.0%, 8.0% and 10.0% ethanol, respectively. When the ethanol concentration was 10%, there was no significant difference (*p* > 0.05) in reducing-sugar-ends content before and after DHPM treatment. Therefore, high ethanol concentration may be used for reduction of polysaccharide degradation during the process of homogenization, which may protect the gelling and emulsifying properties of pectin, which were correlated well with its *M*_w_ [39,40]. In general, the phenomenon of aggregation in the industry is not conducive to homogenization, but in this study, when the ethanol concentration was 10%, the average particle size of pectin was only 2239 (±35) nm, which will not affect the homogenization operation, and will not cause blockage to the homogenizer.

By analyzing the results relating to the average particle size, the average *M*_w_ and the reducing-sugar-ends content, it can be inferred that disaggregation was dominant while degradation was minimized at high ethanol concentration. Antoniou et al. [22] studied the conformation of polymers in a water and polar organic solvent system, and observed that ethanol can increase the density of intermolecular and intramolecular H-bonded networks. With increased concentrations of ethanol, dextran had a tendency to coil and H-bonded networks became rigid, and as a result, dextran was protected from damage. This mechanism may also account for the decrease of DHPM-induced pectin degradation as the ethanol concentration increased from 0% to 10.0%.

### 3.2. Change of Surface Topography

**Figure 4:**
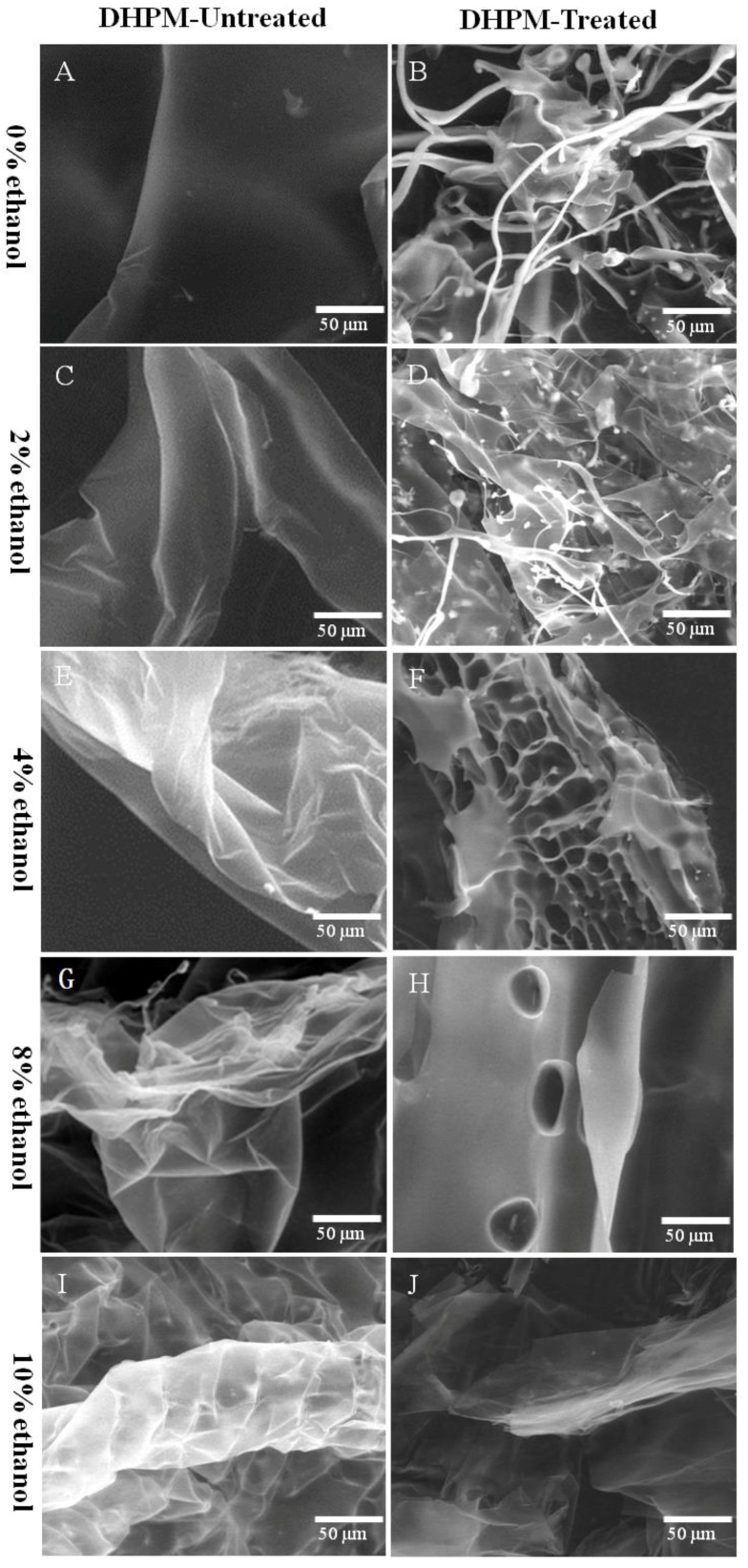
Environmental scanning electron micrograph of pectin treated with or without DHPM under different ethanol concentrations.

The surface topography of pectin obtained through environmental scanning electron microscopy is shown in Figure 4. Without DHPM treatment, it was found that the apparent morphology of pectin contributed to the aggregation or curvature to some extent, and that the degree of aggregation or curvature increased with the ethanol concentration. When the concentration of ethanol reached 10%, an obvious wrinkle was observed (Figure 4I).

After DHPM treatment, the surface topography of pectin was significantly changed. Specifically, when the concentration of ethanol was 0% or 2.0%, the pectin had the filamentous structure (Figure 4B,D). When the concentration of ethanol was 4.0%, there was a honeycomb structure (Figure 4H). When ethanol concentration was 8.0%, the structure of pectin became more compact and there were fewer holes (Figure 4F). When the ethanol concentration reached 10.0%, the sample was dispersed into a lot of flake structures (Figure 4J). These results indicated that the degree of pectin degradation appeared to be lowered with the increase of ethanol concentration, which was consistent with the results of average *M*_w_ and reducing-sugar-ends content. Jiang et al. [41] studied the morphology of pectin (*Ficus awkeotsang*) heated in water and 95% ethanol, and they observed considerably similar morphologies.

### 3.3. Characterization of Possible Reactions

**Figure 5:**
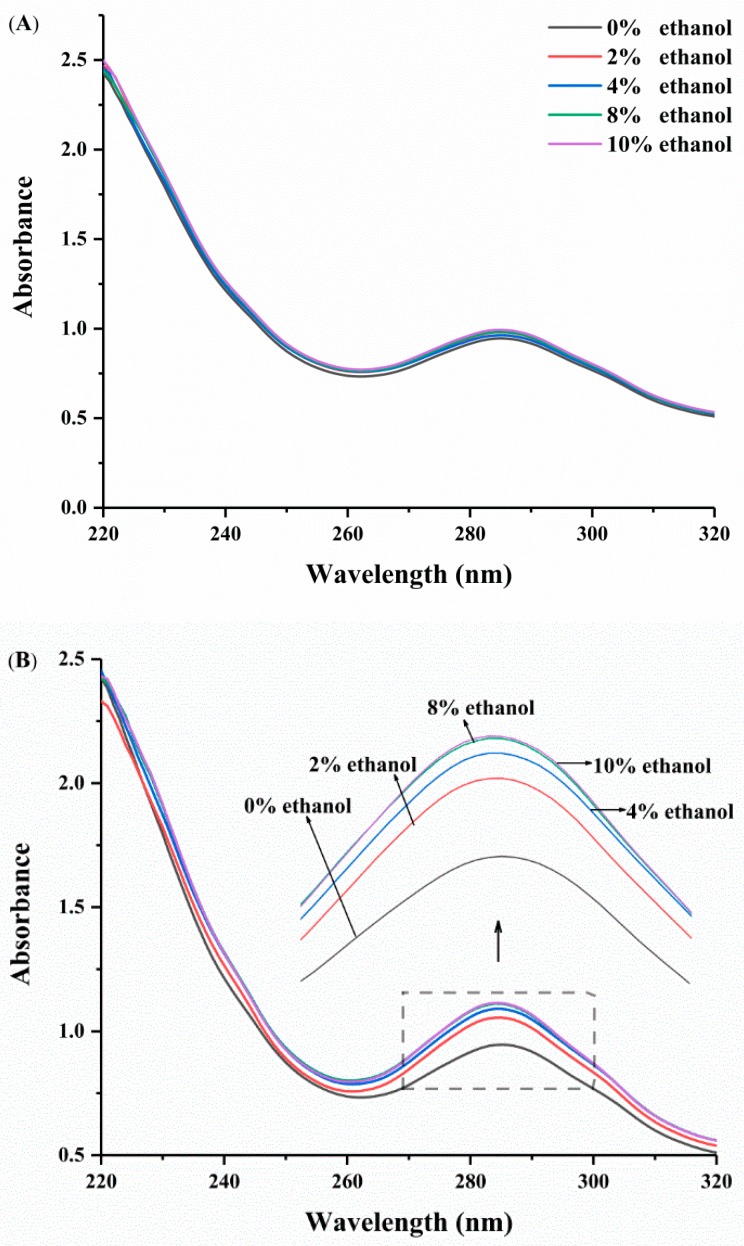
The UV spectra of pectin treated with (**B**) or without (**A**) DHPM under different ethanol concentrations.

To detect the possible reactions involved in DHPM, the UV spectra of the pectin samples were collected. The absorption of the pectin samples at 235 nm was associated with unsaturated uronide formation due to β-elimination [6]. The absorption at 285 nm was attributed to the presence of carbonyl groups in galacturonic acid units [42]. As shown in Figure 5A, there was no change in the absorption intensity at 235 and 285 nm when pectin was dispersed in different ethanol concentrations without DHPM treatment. After DHPM treatment, the peak at 235 nm remained unchanged. This result indicated that β-elimination did not occur in the system. However, the absorption intensity at 285 nm of pectin in the water-ethanol system was discovered to be stronger than that in the pure water system (Figure 5B). This result indicated that samples may have been demethylated during DHPM.

**Figure 6:**
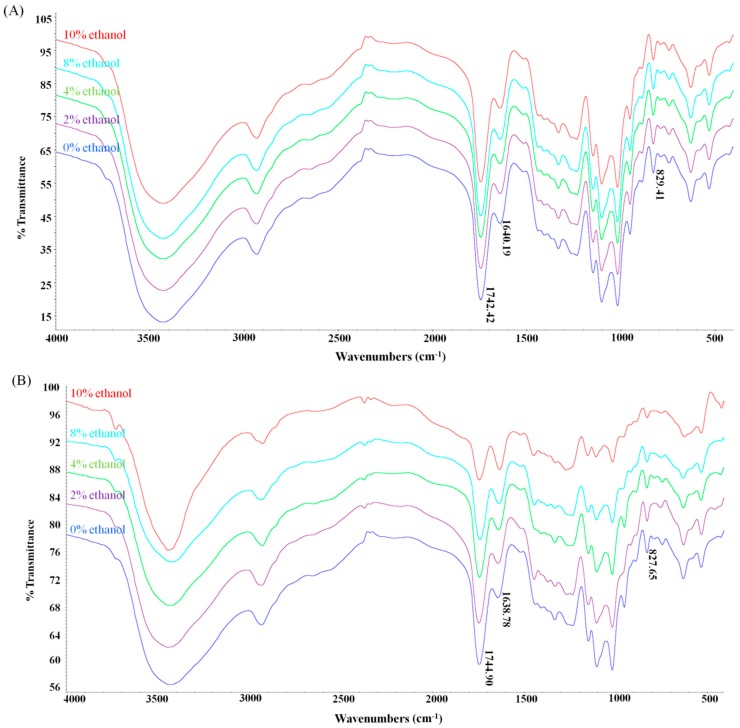
The FT-TR spectra of pectin treated with (**B**) or without (**A**) DHPM under different concentrations.

The FT-IR spectra of pectin before and after DHPM treatment are shown in Figure 6. All pectin samples exhibited similar FT-IR spectra. The characteristic absorption bands exhibited no shift at approximately 3400 cm^−1^ (O–H stretching), 3000–2800 cm^−1^ (C–H stretching and bending), 1744 cm^−1^ (C=O esterified), 1625 cm^−1^ (COO– stretching), 1417 cm^−1^ (COO– stretching) and 1300–1000 cm^−1^ (C=O stretching). The peak areas of the free carboxyl groups and esterified groups are usually used to determine the DM [39]. In our study, the DM of untreated pectin calculated from the data of FT-IR spectra was 67.1%, which was consistent with the result obtained from the titrimetric method. In addition, it was observed that the FT-IR absorption peak of the samples in the water-ethanol system without DHPM treatment remained unchanged (Figure 6A), indicating that no demethoxylation has occurred.

The glycosidic bond spectrum is mainly in the “fingerprint area” around 950–1200 cm^−1^. After DHPM treatment, it was found that the absorption intensity of the 827.65 cm^−1^ peak was weakened. This result indicated that DHPM probably caused a rupture of glycosidic bonds. In addition, the absorption intensity was weakened in the 1744.90 cm^−1^ band but enhanced in the 1638.78 cm^−1^ band (Figure 6B). The DM of DHPM-treated pectin was 67.3%, 66.7%, 63.2%, 60.0% and 58.7% in the presence of 0%, 2.0%, 4.0%, 6.0%, 8.0% and 10.0% ethanol, respectively. These results indicated that demethylation may have occurred when pectin was DHPM-treated in water-ethanol systems. This finding was consistent with the UV results obtained at 285 nm. Shpigelman et al. [19] and Liu et al. [43] also reported that the DM of pectin was decreased after high-pressure homogenization.

We previously reported that DHPM caused a degradation of high-methoxyl pectin by rupturing glycosidic bonds, and the primary structures of pectin were not affected by the DHPM treatment, so that β-elimination and demethoxylation did not occur during DHPM in aqueous solutions [18]. In this study, DHPM caused not only the rupture of glycosidic bonds, but also some extent of demethylation in the water-ethanol system. However, it is unclear why demethylation can occur in the water-ethanol solution instead of the aqueous solution during DHPM. This question requires further investigation.

### 3.4. Mechanism of Pectin Disaggregation and Degradation during DHPM

**Figure 7:**
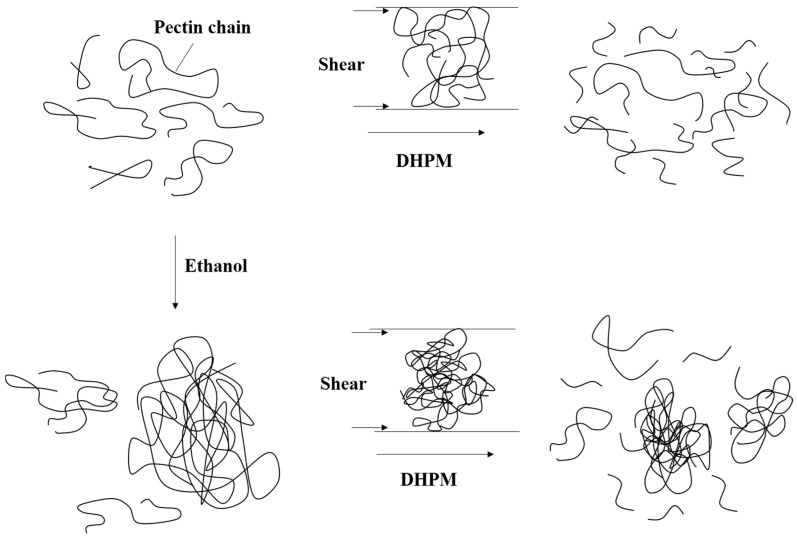
Schematic diagrams to illustrate the disaggregation and degradation process of pectin treated by DHPM in the water-ethanol system.

In our previous study, we proposed a model to illustrate the degradation of pectin induced by DHPM in aqueous solution [18]. Based on the discussion above, a model may be updated to illustrate the disaggregation and degradation process of pectin when it was treated by DHPM under different ethanol concentrations, as shown in Figure 7. Ethanol, as a polar solvent, can compete with pectin in binding water molecules. Pectin molecules come closer to each other and form more hydrogen bonds among themselves, rather than between pectin and water molecules, with the increase of ethanol concentration [21]. This mechanism promotes aggregation of pectin, as reflected by the positive relationship between average particle size and ethanol concentration. When aggregation was formed, fewer chains can be sheared or torn by DHPM treatment. In addition, part of the DHPM-supplied energy was used to disaggregate the long pectin chains, which means that there was less energy supplied for degradation, which is why the change of average particle size of pectin was large, but the changes of average *M*_w_ and reducing-sugar-ends content were minimal when pectin was DHPM-treated at high ethanol concentration. Therefore, both disaggregation and degradation occurred with DHPM treatment at all ethanol concentrations, and disaggregation was dominant at a high ethanol concentration, whereas degradation instead was dominant at a low ethanol concentration.

## 4. Conclusions

When pectin was dispersed in ethanol solutions, the extent of aggregation states was increased, which manifested as an increase of average particle size, decreased amount of reducing-sugar-ends content, but unchanged average *M*_w_. Although pectin could be degraded and disaggregated at all ethanol concentrations during DHPM treatment, the extent of disaggregation increased while the extent of degradation reduced with increasing ethanol concentration, which was shown as the greatest reduction of average particle size but the minimal decrease of average *M*_w_ at an ethanol concentration of 10%. Therefore, a high ethanol concentration may be used for the reduction of pectin degradation during the process of homogenization.
